# Targeting Fibronectin to Overcome Remyelination Failure in Multiple Sclerosis: The Need for Brain- and Lesion-Targeted Drug Delivery

**DOI:** 10.3390/ijms23158418

**Published:** 2022-07-29

**Authors:** Pauline E. M. van Schaik, Inge S. Zuhorn, Wia Baron

**Affiliations:** 1 Section Molecular Neurobiology, Department of Biomedical Sciences of Cells & Systems, University of Groningen, University Medical Center Groningen, Antonius Deusinglaan 1, 9713 AV Groningen, The Netherlands; p.e.m.van.schaik@umcg.nl; 2 Department of Biomedical Engineering, University of Groningen, University Medical Center Groningen, Antonius Deusinglaan 1, 9713 AV Groningen, The Netherlands

**Keywords:** blood–brain barrier, extracellular matrix, fibronectin, liposomes, multiple sclerosis, nanomedicine, oligodendrocytes, PLGA, remyelination, therapeutic targets

## Abstract

Multiple sclerosis (MS) is a neuroinflammatory and neurodegenerative disease with unknown etiology that can be characterized by the presence of demyelinated lesions. Prevailing treatment protocols in MS rely on the modulation of the inflammatory process but do not impact disease progression. Remyelination is an essential factor for both axonal survival and functional neurological recovery but is often insufficient. The extracellular matrix protein fibronectin contributes to the inhibitory environment created in MS lesions and likely plays a causative role in remyelination failure. The presence of the blood–brain barrier (BBB) hinders the delivery of remyelination therapeutics to lesions. Therefore, therapeutic interventions to normalize the pathogenic MS lesion environment need to be able to cross the BBB. In this review, we outline the multifaceted roles of fibronectin in MS pathogenesis and discuss promising therapeutic targets and agents to overcome fibronectin-mediated inhibition of remyelination. In addition, to pave the way for clinical use, we reflect on opportunities to deliver MS therapeutics to lesions through the utilization of nanomedicine and discuss strategies to deliver fibronectin-directed therapeutics across the BBB. The use of well-designed nanocarriers with appropriate surface functionalization to cross the BBB and target the lesion sites is recommended.

## 1. Introduction

Multiple sclerosis (MS) is a neuroinflammatory and neurodegenerative disease that is characterized by the presence of demyelinated lesions. MS is generally diagnosed between the third and fifth decade of life, with women being two to three times more likely to be affected than men [1]. Initially, MS can present itself as a clinically isolated syndrome (CIS) [2] when patients typically face symptoms indicatory of a demyelinating insult to the optic nerve, spinal cord, brainstem, or cerebral hemisphere [1]. A second neurological event converts the CIS to clinically definitive MS. Initially, patients experience spontaneous recovery due to endogenous remyelination of the lesioned area. Most patients with this relapsing–remitting (RRMS) disease course will enter a secondary progressive disease (SPMS) phase in which their disability progressively worsens. A small proportion of patients progressively deteriorate without showing relapses and remissions at first and are classified as primary progressive MS (PPMS) patients [3,4].

The exact cause of MS is yet unknown, though several risk modifiers were identified. Genetic association studies revealed several MS risk gene variants, many of which are involved with immune functioning [5]. Environmental factors include viral infections [6,7], vitamin D deficiency, cigarette smoking, and diet [8]. Current therapies are either symptomatic in nature or rely on immune-modulating strategies, thereby delaying the time and severity of new lesion formation. However, these therapies do not prevent disease progression and often fail in progressive MS patients. Progressive axonal loss is key to the continuous and irreversible neurological decline in progressive MS [9]. In addition to ensuring saltatory conduction, oligodendrocytes secrete via myelin metabolic and trophic factors that maintain the integrity and survival of axons [10,11]. Therefore, next to primary axon damage, a major cause of axonal loss in chronic stages of MS is secondary axon degeneration because of remyelination failure [12]. Hence, to halt disease progression, the development of treatments that preserve axons, i.e., via the promotion of remyelination, is an essential therapeutic goal.

Oligodendrocytes (OLGs) are responsible for myelinating neuronal axons in the CNS and mature from oligodendrocyte progenitor cells (OPCs). Remyelination in MS lesions is often insufficient despite the presence of OPCs and/or surviving mature OLGs in most lesions [13,14]. Therefore, therapeutic interventions must overcome the pathogenic MS lesion environment. Perturbed remodeling of the extracellular matrix (ECM) in MS lesions likely plays a causative role in remyelination failure [15,16]. In this review, we focus on the ECM protein fibronectin (Fn) that, in its aggregated form, persists in MS lesions and impairs OPC differentiation and remyelination [17,18]. We outline the beneficial roles of Fn in the neurovascular unit and the detrimental roles of Fn in MS pathology and discuss therapeutic strategies and agents to prevent Fn aggregation and/or to overcome Fn-mediated inhibition of remyelination. In addition, to translate these therapeutic strategies for myelin regeneration to the clinic and consider the beneficial role of Fn in the neurovascular unit, we discuss the need and strategies for the brain- and lesion-targeted delivery of nanomedicine.

## 2. Multiple Sclerosis: An Unmet Need for a Remyelination-Based Therapy to Stop Disease Progression

MS has long been perceived as an autoimmune disease mediated by autoreactive T and B cells, which is an ‘outside-in hypothesis’ that has been substantiated by successful disease-modifying immunomodulatory therapies [19]. Demyelinating plaques arise due to an autoimmune response against myelin, mediated by CD8+ and CD4+ T cells, called autoimmune encephalomyelitis [20]. Oligoclonal bands detected in the cerebrospinal fluid (CSF) simultaneously indicate the presence of immunoglobulin-producing B cells [21]. Especially in the early stages of RRMS, immunomodulatory therapies show high efficacy, indicating that inflammation appears to play a crucial role in disease development [3,22]. Alternatively, intrinsic neuronal or glial disturbances may initiate a cascade of inflammation, which is coined the ‘inside-out hypothesis’ [3,9]. This is corroborated by the finding that brain atrophy manifests early and is a more important determinant of disease progression than lesion load. Additionally, brain atrophy in PPMS may exceed that observed in RRMS [23]. Furthermore, due to the low efficacy of immunomodulatory therapies in the progressive phase of MS, alternative disease mechanisms appear to play a role in disease progression.

While remyelination is known to occur in the early stages of the disease, this regenerative capacity of the CNS diminishes with age, ultimately leading to the accumulation of permanently demyelinated lesions and aggravated clinical disability [24]. In RRMS, new lesions form during relapses, which in the white matter are characterized by inflammation and blood–brain barrier (BBB) damage. MS lesions display profound heterogeneity, leading to the development of several lesion classification systems, each of which focuses on different aspects of the lesion stage and activity. Kuhlmann et al., proposed an updated classification system of demyelinated white matter lesions based on the presence and distribution of macrophages/microglia, resulting in the classification of active, mixed active/inactive, or inactive lesions [25]. Typical actively demyelinating lesions as observed in RRMS are not commonly seen in PPMS, though it was recently shown that a large majority of PPMS patients had mixed active/inactive lesions [26]. This indicates that ongoing demyelination and inflammation may be ubiquitously present. In addition, chronic diffuse inflammation of (normal-appearing) white matter and cortical demyelination are frequently observed in progressive MS [27,28,29]. Cortical lesions are classified based on location and are less associated with the infiltration of immune cells, as is generally seen in actively demyelinating lesions of the white matter [30]. The amount of intracortical and leukocortical lesions shows a strong correlation with clinical impairment [26]. For example, a substantial proportion of (progressive) MS patients develops a form of cognitive impairment, which strongly correlates with cortical demyelination [31,32]. Particularly, reductions in information processing speed, working memory, and executive functioning are reported [33,34], which reflect deficits in frontal lobe functioning [35]. Furthermore, a direct link between white matter lesion volume and cognitive impairment in early-stage MS was recently reported [36]. Due to the functional relationship between demyelinating lesions and clinical symptoms, remyelination can ameliorate clinical symptoms, including cognitive and physical functioning [37]. Indeed, post-mortem inspections of brain lesions demonstrated that MS patients with a higher load of remyelinated lesions had lower clinical disability scores [26,38].

Remyelination of lesioned areas occurs, causing the appearance of so-called ‘shadow plaques’, or partly remyelinated areas at the border of lesions [25]. Remyelinating areas are more pronounced in active than in inactive lesions and are hardly present in mixed active/inactive lesions [14]. Nevertheless, only a small proportion of lesions fully remyelinate, which is a process that negatively correlates with the amount of TMEM119+ and iNOS+ (indicative of an inflammatory phenotype) myeloid cells present in the lesion [14] and diminishes with age and disease chronicity [39]. Thus, demyelination and subsequent remyelination are two antagonistic processes in which inflammatory and neurodegenerative processes concurrently contribute to the development and maintenance of lesions. Next to mitigating the excessive inflammatory response promoting demyelination, strategies aimed at enabling remyelination may contribute to alleviating the disease burden [3,40].

## 3. Remyelination Failure in MS: Perturbed ECM Remodeling in White Matter Lesions

Recent evidence has demonstrated that pre-existing surviving mature OLGs retain their myelinating capacity after a demyelinating insult [41]. Moreover, remyelination in shadow plaques can mainly be attributed to pre-existing mature OLGs rather than newly differentiating OPCs [42]. Nevertheless, OLGs surviving a demyelinating insult in zebrafish were shown to make few and not-well-targeted myelin sheaths, which is a finding that corresponds to observations in remyelinated MS lesions [43]. In contrast, OPC-based remyelination, i.e., the generation of newly formed OLGs, is more efficient, as evident in experimental models of demyelination. OPCs are present in the adult brain throughout the lifespan and preserve the capacity to differentiate into mature OLGs [13]. The absence of pro-oligodendrogenic factors and the presence of anti-oligodendrogenic factors in MS lesions were postulated as complicit elements in preventing OPC maturation [44]. Indeed, most chronically demyelinated lesions contain OPCs that apparently fail to differentiate and mature [45,46,47,48], while in a subset of mixed active/inactive lesions, few OPCs are present, which is likely due to cellular expression of chemorepellent factors [47]. Both cell-intrinsic factors, such as the maturation and differentiation stages of OLG lineage cells [49], and cell-extrinsic factors, such as the composition of the ECM, determine the inhibitory milieu at the injury site [50]. Moreover, other inhibitory factors present in the lesioned area, such as infiltrating lymphocytes and inflammatory mediators, oxidative stress, and irreversible damage to OPCs and OLGs, may all be conducive to the non-permissive milieu [51,52].

General aging has been hypothesized to play an important role in the reduced regenerative capacity of the brain [39,53,54], as age is also the best predictor of disease progression [55,56]. In rats, remyelination following toxin-induced demyelination slowed down with age [57] due to an impairment in OPC recruitment and differentiation [53]. Inefficient epigenetic downregulation of OLG genes that inhibit OPC maturation may underlie this [58], as well as a failure of OPCs to respond to pro-differentiation signals with aging [54,59]. Whether the intrinsic aging of OPCs or the aging environment induces these deficits has been elusive, though recent data hint at a significant role of environmental cues, namely, a gradual stiffening of the extracellular microenvironment and general brain tissue stiffening may contribute to OPC malfunctioning over time [60]. Thus, OPCs increasingly lose their capacity to proliferate, migrate, and differentiate with age, likely due to a non-permissive stiffened aging environment. In situations where demyelination is exacerbated, as in MS, this poses an increased risk as OLGs surviving demyelinating insults and OPCs also exhibit diminished myelinating capacities and face additional disease-specific alterations in stiffness [60].

The composition of the ECM is a major determinant of tissue stiffness. The ECM restrains the movement of cells by forming a physical scaffold and is simultaneously important for maintaining healthy brain homeostasis by directing cell differentiation, growth, and migration [61,62,63]. It is composed of an interactive network of fibrous-forming proteins, such as collagens, elastin, Fn, laminins, glycoproteins, proteoglycans, and glycosaminoglycans. Cells receive and integrate signals from the ECM via specified surface receptors with an affinity for one of the ECM constituents [63]. Concurrently, signaling molecules, such as growth factors, cytokines, and chemokines, can be stored temporarily within the matrix and released when needed, meaning the cells and the extracellular milieu form a bi-directional synergy [63,64]. In turn, cytokine release (i.e., TFG-β, TNF-α, and IFN-γ) during inflammation can affect ECM synthesis and turnover, thereby causing changes in the ECM composition [16,65]. In particular, due to their repeating glycosaminoglycan (GAG) chains, proteoglycans have the ability to bind cytokines and growth factors [66]. For example, heparan sulfate chains are known to bind basic fibroblast growth factor (bFGF) [67]. Furthermore, fibronectin domains were found to bind growth factors, in particular vascular endothelial growth factor (VEGF) [68] and hepatic growth factor [69]. Subsequent proteolytic cleavage of ECM proteins can result in a directed release of these factors in the extracellular milieu, thereby contributing to local cell differentiation and proliferation [66].

The interaction between ECM and OLG lineage cells decides whether remyelination can occur based on ECM rigidity and activation of intracellular signaling pathways. A softer matrix inhibits cell differentiation and myelination, while gliosis is stimulated by a stiff matrix [70,71]. OPCs are mechanosensitive [72] and in vitro data suggest that a relatively stiff matrix favors OPC proliferation and differentiation, while a soft matrix is beneficial for myelination [73]. Distinct ECM proteins differentially affect OPC differentiation. While Fn induces OPC proliferation and impedes OPC differentiation [17,74], laminin stimulates the expression of mature OLG markers and myelin components, including myelin basic protein (MBP) and proteolipid protein (PLP) [75,76,77]. Hence, changes in the ECM protein composition modulate reparative processes by allowing for altered cell behavior, be it beneficial or detrimental [15]. In acute demyelinating conditions, a transient change in ECM components occurs, which consists of increased tissue stiffness that reverses upon remyelination. In chronic demyelinating conditions, these changes are not reversed and are accompanied by enhanced ECM deposition [78]. This demonstrates that an adequate response to acute changes in the lesioned area is stalled in areas of chronic demyelination. However, the transient deposition of ECM proteins is a natural response to CNS injury, particularly the deposition of chondroitin sulfate proteoglycans (CSPGs) and Fn at the lesion [50,79]. Astrocytes are the main source of Fn and CSPGs in the CNS [80], which form a glial scar at the lesioned area through a process of reactive astrogliosis [81]. CSPGs and Fn deposited in demyelinated lesions aid OPC recruitment but impair OPC differentiation and myelination, indicating that timely Fn and CSPG removal is required for efficient remyelination to occur [17,82,83,84].

Hence, the composition of the ECM and perturbed remodeling during inflammatory and demyelinating insults typically hampers the establishment of a remyelination-permissive milieu. Theoretically, this implies that ECM-mediated inhibition of OPC differentiation in MS lesions may be therapeutically targeted by degradation enzymes that remove the inhibitory ECM proteins. Indeed, enzymatic digestion of CSPGs with simultaneous supplementation of growth factors aids OPC differentiation and migration after injury [85,86]. However, as CSPGs are also components of the interstitial ECM in the healthy adult brain and Fn is also a component of the BBB BM, targeted delivery of ECM-degradation enzymes to MS lesions is a prerequisite to avoid unwanted side effects. Alternatively, as shown for CSPGs, blocking the transient deposition of ECM proteins upon injury and interfering with ECM-mediated signaling appear to be feasible approaches to prevent or overcome impaired OPC differentiation [87,88,89,90,91]. Nevertheless, the complete absence of an ECM-remodeling response does not necessarily benefit healthy regeneration. Before discussing brain- and lesion-targeted delivery approaches for remyelination therapeutic agents, we first present an overview of the beneficial roles of Fn at the BBB and detrimental roles of Fn in MS pathology and provide potential therapeutic agents to overcome Fn-mediated remyelination failure. For CSPG-targeting approaches to promote remyelination in MS, we refer to excellent recent reviews [92,93].

## 4. Fibronectin: Multifaceted Roles in the CNS and in the Pathogenesis of MS

The Fn gene (*FN1*) contains three repeating domains (I, II, and III), each of which can be found in other molecules, indicating that Fn evolved through exon shuffling [94,95,96]. Though Fn is transcribed from a single gene, 20 different human splice variants are known, suggesting that the protein has a multitude of functions depending on the splicing of the pre-mRNA [95]. Broadly speaking, Fn is present in plasma and body fluids in its soluble dimeric form, while an insoluble variant with a cellular origin can be found in the ECM of tissues. Cellular Fn exclusively expresses extradomain A (EDA, EIIIA in rodents) and/or EDB (EIIIB in rodents) and shows higher heterogeneity than plasma Fn due to its role in the ECM modeling of different tissues [96]. Fn is a ligand for integrins of the β1, β3, β5, and β6 families [95,96,97,98]. Integrins are cell surface heterodimers composed of an α subunit noncovalently linked to a β subunit. Of these, about 20 heterodimers are known, each of which binds to specific ligands [99]. They form a physical link between the ECM and the cytoskeleton of cells, thereby allowing for the transduction of extracellular signals [100,101] and the control of cell behavior.

### 4.1. Fibronectin and Its Role in BBB Functioning

**Figure 1:**
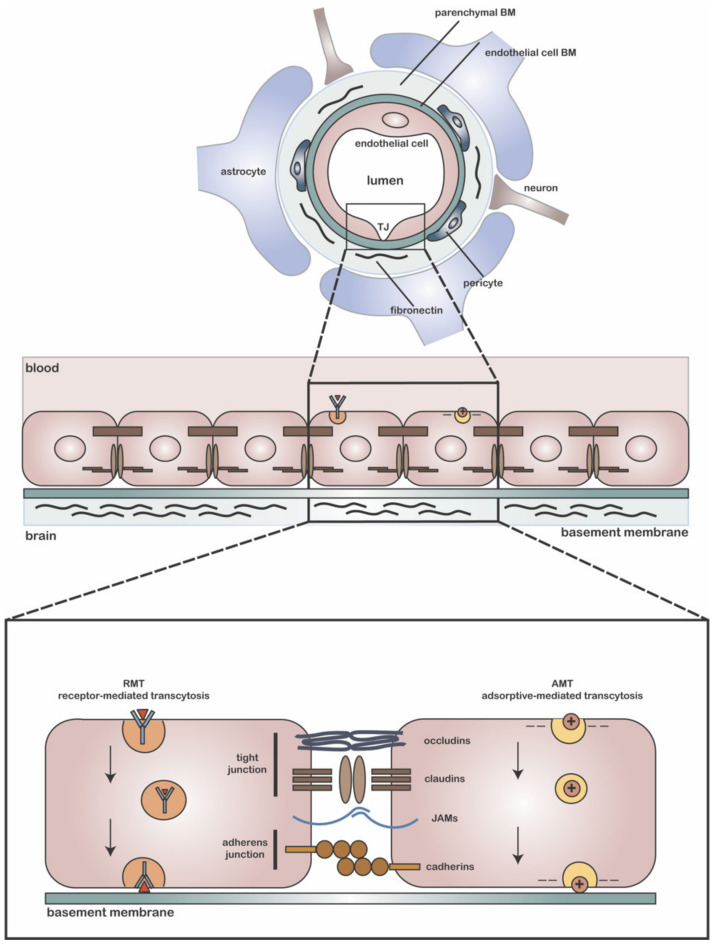
Schematic representation of the blood–brain barrier and active transport mechanisms. The blood–brain barrier (BBB) is formed by a monolayer of specialized endothelial cells, which form together with pericytes, astrocytes, and the basement membrane (BM) to create the neurovascular unit. The BM is a thin sheet of supporting extracellular matrix, including fibronectin, and is composed of endothelial BM and the astrocyte-derived parenchymal BM. Endothelial cells are tightly connected via tight and adherens junctions, which prevent the paracellular passage of molecules. Active transport mechanisms across the BBB include receptor-mediated transcytosis (RMT) or adsorptive-mediated transcytosis (AMT). RMT involves ligand–receptor binding, followed by endocytosis of the receptor complex, intracellular trafficking, and exocytosis at the basal membrane [114]. Conversely, cationic molecules can interact with the negatively charged membrane, thereby inducing transcellular transport of the positively charged molecule. See the text for more details. JAM—junction adhesion molecule; TJ—tight junction.

In the healthy adult brain, the presence of Fn is restricted to the BBB. The BBB consists of a layer of tightly connected endothelial cells that line the brain capillaries and play an important role in maintaining brain homeostasis (Figure 1). The polarized brain endothelial cell monolayer differentially harbors lipids and proteins at its luminal (blood-side) and abluminal (brain-side) membrane [102]. For BBB endothelial cells to maintain their tight barrier function, close contact with astrocytes and pericytes is necessary. Perivascular astrocytic end feet make up the glia limitans, which fully cover the BBB endothelial cells and part of the pericytes. Microglial processes can enter through interspersed slits in the glia limitans, thereby allowing for direct contact with the endothelial basal lamina. This interface is important for controlling water and ion exchange between blood and the brain [103]. The basal lamina, also known as the basement membrane (BM), is a sheet-like ECM structure that provides cells with an adhesive substrate to grow and migrate on and allows for the modulation and transmission of intracellular signals and mechanosensitive physical cues [99,104]. The BBB BM is composed of two parts: an endothelial BM that lines the vascular wall, and one that forms the parenchymal BM of the glia limitans, which is produced by astrocytes (Figure 1) [105,106,107]. The two parts combine to form a protein network that contributes to the maintenance and barrier tightness of the endothelial BBB [61]. In healthy conditions, the parenchymal BM protects the brain parenchyma from leukocyte infiltration, while in inflammatory disease states, its degradation by matrix metalloproteinases (MMPs) disrupts this function [108]. Pericytes, which are believed to derive from migrating mesenchymal cells, neural crest cells, or macrophages [109,110,111,112], are embedded in the BM of the BBB, where direct exchange of cellular signals is mediated by GAP junctions. Hence, the BBB is not solely formed by tightly connected endothelial cells but also consists of endothelial cells, astrocytes, pericytes, and an ECM, which is referred to as the neurovascular unit (NVU) [113].

The main components of the BM are Fn, laminin, and collagen type IV [115], which are tethered to nidogens and proteoglycans [116,117]. As the composition of the BM plays a pivotal role in providing endothelial cells with a supportive growth substrate, changes in the BM protein composition induce functional changes in endothelial cell phenotypes [118]. The importance of the supporting cell types for BBB integrity is highlighted by the lower endothelial barrier integrity and an altered cytoplasmic anchoring of tight junctions (TJs) in monocultures [119] compared with co-cultures [120,121]. Indeed, endothelial cells grown on an ECM produced by astrocytes and pericytes show enhanced barrier impermeability compared with cells grown on non-astrocyte/pericyte-derived ECM [122]. A pericyte-derived ECM most rapidly increased the barrier resistance of endothelial cells, induced stronger expression of the TJ proteins occludin and claudin-5, and contained the largest relative amount of Fn, indicating that Fn is essential for proper barrier induction [61]. The Fn-binding integrins α5β1 and αvβ3 induce proliferation and aid the survival of BBB endothelial cells via the MAP kinase signaling pathway [123], while on laminin endothelial cells enter growth arrest on laminin via integrin α2β1 [124]. Alongside regulating interactions with ECM components, integrins organize the proper functioning of BBB-specific molecules. For example, VE-cadherins form the molecular basis of adherens junctions that regulate the permeability of the BBB endothelium. Like integrin receptors, VE-cadherins are able to transduce extracellular signals and coordinate cell attachment and migration [106]. Fn treatment of endothelial cell layers can disrupt VE-cadherin-mediated cell–cell interaction through interaction with integrin αvβ3 [125]. Direct activation of integrin αvβ3 similarly disrupts the endothelial monolayer integrity by mislocalizing VE-cadherins [126]. These results demonstrate a direct link between endothelial–ECM interactions and functional properties of BBB endothelial cells, as reviewed in [127].

**Figure 2:**
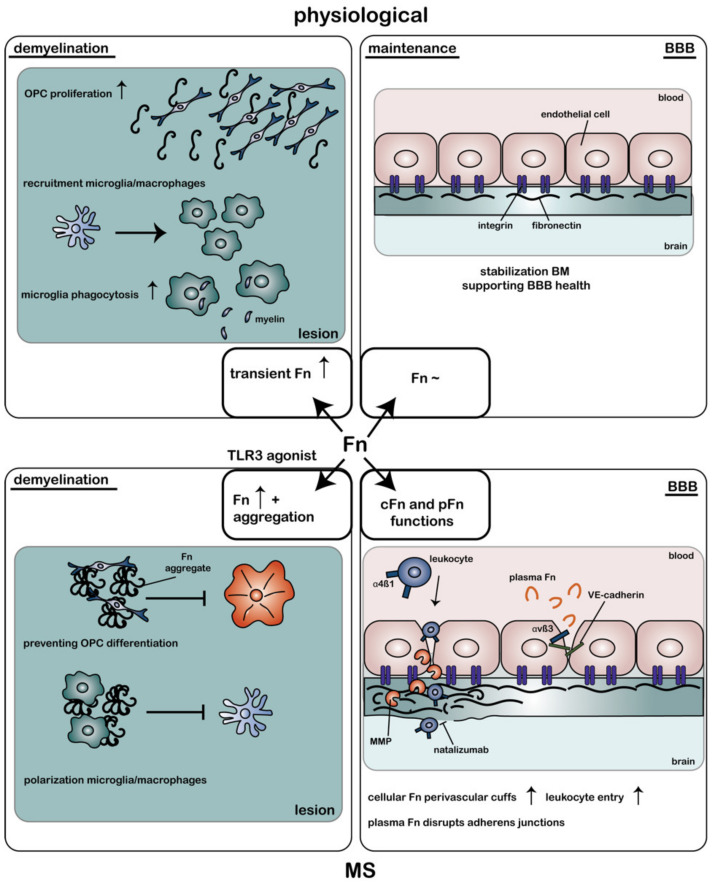
Role of fibronectin in the physiological adult brain, upon demyelination, and in multiple sclerosis. In the non-injured adult brain, fibronectin (Fn) expression is limited to the basement membrane (BM) of the blood–brain barrier (BBB), stabilizing the BM, thereby supporting BBB maintenance. Upon demyelination in the non-MS brain, Fn expression in the parenchyma is transiently upregulated, which aids (1) oligodendrocyte progenitor cell (OPC) proliferation in the lesioned area, (2) activation and recruitment of pro-inflammatory microglia and macrophages, and (3) myelin debris removal via phagocytosis. Conversely, upon demyelination in the MS brain, Fn expression persists and aggregates under the influence of Toll-like receptor 3 (TLR3) activation. Fn aggregates (1) impede remyelination by preventing OPC differentiation and (2) inhibit the switch from a pro-inflammatory to anti-inflammatory phenotype in microglia and macrophages. In addition, in an MS BM, Fn accumulates in perivascular cuffs near the BBB, which aids leukocyte transmigration across the BBB via integrin α4β1–Fn interaction. Natalizumab, which is a clinically approved MS medication, stops leukocyte entry into the CNS by blocking α4-integrins. Furthermore, several matrix metalloproteinases (MMPs) are upregulated in MS, which degrade Fn and other BM constituents, contributing to BBB destabilization and leukocyte entry into the brain parenchyma. Furthermore, in MS, plasma Fn (pFn) may interact with integrin αvβ3 expressed on endothelial cells, thereby destabilizing VE-cadherins and increasing the BBB permeability. Upward arrow indicates ‘enhanced’. cFn—cellular fibronectin; pFn—plasma fibronectin; VE-cadherin—vascular endothelial-cadherin.

The altered composition of ECM proteins surrounding the BBB in disease states plays a causative role in disturbing barrier integrity in several neurological diseases. In MS, the BBB becomes disrupted, meaning the barrier’s tightness is reduced, followed by an increase in leakiness [106]. In addition, monocytes and T cells cross the BBB endothelium, after which they gain access to the vascular and astroglial BM and brain parenchyma [128]. MMPs are known to degrade the ECM surrounding the blood vessels, thereby allowing for leukocyte infiltration into the CNS [129,130]. Furthermore, adhesion molecules, such as ICAM-1, act as receptors for leukocytes and are upregulated in MS lesions [131], thereby facilitating their migration across the BBB [132]. In active and mixed active/inactive lesions, mononuclear cells accumulate between the astroglial and vascular BM, which leads to a widened perivascular space and the appearance of so-called perivascular cuffs [107]. The ECM of these perivascular cuffs has a differential composition, with fiber-like networks containing several laminin isoforms, Fn, collagen IV, and heparan sulfate proteoglycans. This diverging composition may aid the migration of leukocytes [107]. Astrocytes are likely a source of the altered ECM in the BM, including extracellular Fn [133], and the expression of a splice variant of Fn that binds integrin α4β1 may facilitate leukocyte migration into the CNS [134] (Figure 2). Blocking α4-integrins indeed inhibits leukocyte infiltration in EAE and alleviates clinical symptoms [135,136]. Natalizumab, which is an antibody against α4-integrins used in the treatment of MS and Crohn’s disease, similarly blocks peripheral leukocyte trafficking to the CNS and was shown to be highly effective in preventing relapses in RRMS [137,138]. Nevertheless, natalizumab treatment carries the substantial risk of developing progressive multifocal leukoencephalopathy (PML) with long-term treatment [139].

### 4.2. Fibronectin as a Vasculogenic Regulator in MS

During angiogenesis new blood vessels are being formed, which requires signaling from growth factors, e.g., vascular endothelial growth factor (VEGF) and bFGF [140,141], and the ECM through integrins [123,142,143]. Fn was found to promote cell survival, proliferation, and migration of endothelial cells [123,144,145,146], while laminin induces differentiation and stabilization [123,147,148]. Thus, high expression levels of Fn are required during the formation of new blood vessels to support endothelial cell proliferation, while the presence of laminin is essential for their maintenance. Alterations in vasculature properties contribute to disease development and maintenance in experimental models of MS. For example, in EAE, VEGF is upregulated in the spinal cord during relapses and is correlated to demyelination and cell infiltrate levels [149,150]. An increase in vascular density (neo-angiogenesis) may be a regenerative response to hypoxic conditions [151,152], but can eventually lead to the formation of abnormal, leaky blood vessels (pathological angiogenesis). Angiogenesis under hypoxic conditions is (at least partly) regulated by Fn–integrin α5β1 interactions [153]. In EAE, an increase in the number of blood vessels in the white matter of the spinal cord is observed during the pre-symptomatic phase of the disease, which is accompanied by an elevation in Fn and α5β1 levels. This indeed reinforces the notion that α5-integrins mediate endothelial cell proliferation, thereby bolstering the formation of new blood vessels in EAE [154]. However, it is unclear whether this temporal increase in angiogenesis is mainly detrimental or beneficial. In MS, an increase in blood vessels is reported at lesions [155], indicating angiogenic remodeling. In addition, MS lesions have increased vessel expression of Fn, which correlates with the degree of inflammation [156]. Indeed, while plasma Fn levels are low under homeostatic conditions, they sharply increase during episodes of heightened inflammation, such as those observed during MS relapses and acquired vascular damage [157,158]. Whether this increase in plasma Fn or enhanced expression of cellular Fn by endothelial cells or astrocytes and deposition in the BMs contributes to angiogenesis remains to be determined.

### 4.3. Fibronectin (Aggregates) as a Remyelination Inhibitor in MS

In the adult brain, Fn expression in the parenchyma is very low. However, transient Fn expression by resident cells is a common response to tissue injury [159]. Several studies similarly reported a transient upregulation of Fn in toxin-induced demyelinated CNS lesions, which declined at the onset of remyelination of the lesioned area [17,84,160,161]. The source of this interstitial Fn could partly be attributed to nearby cellular Fn-producing astrocytes [17,74]. In addition, the reduction in BBB integrity in MS results in blood proteins, including plasma Fn, gaining access to the brain parenchyma [162] and implicates BBB breakdown as one of the primal factors in disease onset [162,163,164]. The appearance of Fn in demyelinated lesions has a bifold effect on OPCs (Figure 2). Fn promotes proliferation and migration of OPCs via integrins αvβ3 and αvβ1, respectively [74,165,166,167], while Fn hinders the maturation of OPCs into fully differentiated OLGs and the formation of new myelin membranes [168,169,170]. The latter may be beneficial by allowing first for myelin debris removal and OPC recruitment to the lesion. Degradation-resistant Fn aggregates are observed in inflammation-mediated demyelination, including at the relapse phase in EAE and in chronic demyelinated MS lesions. The persistent presence of Fn (aggregates) in MS lesions impedes myelin biogenesis in a β1-dependent manner [169], which is an effect that is dominant over laminin-2-mediated positive signals for myelin biogenesis [171]. Therefore, the presence of Fn (aggregates) in lesions explains why spontaneous regeneration in MS does not occur (Figure 2). This hypothesis was confirmed by studies showing that intralesional injection of Fn aggregates into toxin-induced demyelinated lesions inhibits OPC differentiation and remyelination [17,18]. Moreover, remyelinated lesions contain hardly any Fn aggregates [17].

Microglia are innate immune cells specific to the brain, where they exist as distinct subtypes that are involved in several regulatory functions. These include the removal of remyelination-inhibiting myelin debris [172,173,174] after injury in conjunction with infiltrated peripheral-derived macrophages [175,176] and the generation of pro-regenerative signaling factors [177]. In fact, while a pro-inflammatory response is initially required to remove myelin debris [178], a switch to a more regenerative phenotype of microglia is required for remyelination to proceed [179]. This illustrates how the presence of an inflammatory response in MS is not unequivocally deleterious [180]. Microglia and macrophages are located in MS lesions and express the appropriate receptors for binding Fn [181,182,183]. Therefore, Fn aggregates in MS lesions may not only influence the behavior of OPCs, but also that of microglia and macrophages (Figure 2). Indeed, microglia and macrophages that are grown on aggregated Fn adopt an activated phenotype consisting of an amoeboid morphology and the expression of both pro- and anti-inflammatory markers [184]. If the pro-inflammatory phenotype of microglia and macrophages in the lesion is sustained, this may impair the subsequent remyelination process [185]. Of relevance, immunohistochemical analysis demonstrated that microglia and macrophages in MS lesions still display pro-inflammatory markers [14,186,187]. Remarkably, and in contrast to Fn’s effect on OPCs, soluble dimeric (plasma) Fn and aggregated Fn differentially affect microglia and macrophages. The enhanced expression of Fn during an inflammatory insult increases the expression of integrins α4β1 and α5β1, activates microglia [181], and increases the expression of MMP9 [188]. The β1-integrin-induced proliferation of microglia is regulated by cAMP-dependent PKA signaling, which plays a negative regulatory role in β1-integrin translocation [189]. By doing so, Fn plays a role in the activation and recruitment of microglia to the inflamed area (Figure 2). Simultaneously, soluble Fn containing the EIIIA domain was demonstrated to stimulate inflammatory processes through TLR4 activation [190,191], thereby stimulating microglia phagocytosis [192], migration, and proliferation [193]. As such, the appearance of soluble Fn plays a beneficial role in regenerating the lesioned area. Nevertheless, aggregated Fn does not bind TLR4, and its effect on microglial activation is β1-independent [184]. Thus, aggregated Fn differentially affects the lesion environment from soluble Fn and does not aid lesion regeneration. This shows that for successful remyelination, an upregulation of α5-integrins and soluble Fn may initially be important, as these signal to microglia to remove myelin debris present at the lesioned site.

Taken together, in the adult brain, Fn plays a significant regulatory role in (1) maintaining BBB integrity, (2) increasing angiogenesis upon injury, and (3) initial OPC and microglia recruitment to demyelinated lesions. However, its aggregation in MS lesions results in a gain of function, as its aggregated form is resistant to degradation and is an impeding factor in OPC maturation and the sustained presence of pro-inflammatory microglia within MS lesions (Figure 2). Given that Fn signaling to OPCs is dominant over laminin-mediated signals [171], we next discuss therapeutic strategies to specifically overcome Fn-aggregate-mediated inhibition of OPC differentiation to overcome remyelination failure.

## 5. Promoting Remyelination in MS: Therapeutic Strategies to Overcome Fn-Mediated Inhibition of Remyelination Failure

Fn aggregates impair OPC differentiation and remyelination, either directly, or indirectly via Fn-mediated microglia and macrophage dysfunction. Accordingly, a therapeutic benefit will be achieved by counteracting the negative signals of Fn aggregates (Table 1). One strategy to stimulate remyelination in chronically demyelinated lesions is by utilizing factors that can aid OPC differentiation in the presence of aggregated Fn, i.e., blocking or bypassing signals from Fn aggregates to cellular receptors on OPCs and microglia. For instance, exposure to ganglioside GD1a stimulates OPC differentiation, maturation, and myelination in cuprizone-induced demyelinated lesions that contain externally injected Fn aggregates [18]. Although the underlying mechanism remains to be determined, the effect of GD1a is evoked by a PKA-mediated signaling pathway and is mimicked by increasing cAMP levels [18]. Theoretically, agents that increase or prolong cAMP levels, such as PDE inhibitors that are beneficial in experimental models of MS and/or are currently used in clinical trials [194,195,196,197], may overcome Fn-mediated inhibition of OPC differentiation, and thus, benefit remyelination. On the other hand, agents that modulate intracellular signaling pathways may induce unwanted side effects in other cell types, such as microglia and neurons in healthy and injured tissue. GD1a’s effect on OPC maturation and differentiation is, however, (cell-type) specific and only effective in an Fn-containing environment [18], making it a promising therapeutic agent for the treatment of chronically demyelinated MS lesions.

Another strategy to overcome remyelination failure is to prevent Fn aggregate formation, by allowing for timely Fn degradation. The formation of Fn aggregates is associated with an inflammatory process and encompasses disrupted Fn fibrillogenesis [198]. The assembly of fibrillar Fn into a network of high molecular weight fibrils is mediated at the astrocyte surface by integrin α5β1. Soluble Fn dimers bind the receptor, assemble into high-molecular-weight Fn, and self-associate using non-covalent bonds [199,200,201]. As stated before, *FN1* has many splice variants, of which some have specific relevance to fibrillogenesis [202]. These include variants containing cellular Fn-specific EIIIA and/or EIIIB domains. The relative abundance of either domain changes the conformational shape of Fn, thereby altering its function [203,204,205,206,207]. A ‘double inflammatory hit’ mechanism induces Fn aggregation and involves an initial exposure of astrocytes to pro-inflammatory cytokines that are associated with a demyelinating event, resulting in altered Fn splicing and a relative upregulation of the EIIIA-containing Fn [198]. A subsequent hit with a TLR3 agonist interferes with Fn cell-surface binding, thereby increasing Fn aggregation [198]. The degradation of myelin can result in the release of endogenous TLR3 agonists, such as stathmin [208], which is upregulated in myelin obtained from MS lesions [209]. Thus, timely treatment with factors that interfere with Fn splicing and/or TLR3 signaling in astrocytes may preclude Fn aggregation [198].

A possible reason for the absence of Fn aggregates in experimental toxin-induced demyelination models is not only the absence of a combination of BBB disturbances and a prolonged inflammatory component in toxin-mediated demyelination but also the efficient clearance of Fn before it has the chance to aggregate. Due to the efficient clearance of myelin debris and Fn in toxin-induced demyelination models, the likelihood of encountering a TLR3 agonist is small, hence Fn aggregates are unlikely to form during Fn fibrillogenesis. Simultaneously, the upregulation of MMPs, which are endogenous proteinases able to digest ECM components, during the earliest phase of toxin-induced demyelination may aid in the timely removal of Fn [210,211]. In chronic MS lesions, a lack of MMP7 activity may underlie the impairment in Fn clearance [211], thereby increasing the possibility of TLR3-mediated Fn aggregation due to prolonged inflammation and inefficient myelin debris clearance [198]. In addition, MMP7 is pivotal in cleaving aggregated Fn [211]. MMP7 and MMP3 are increasingly expressed in actively demyelinating MS lesions [212,213,214], implying their natural upregulation after a demyelinating insult, while MMP7 is absent in inactive MS lesions [211]. This data hints at the possibility of utilizing MMPs, particularly MMP7, as a relevant therapeutic target for MS to clear Fn (aggregates). Thus, a locally induced upregulation of MMP7 could prepare the lesioned area for subsequent remyelination, mainly by aiding the removal of aggregated Fn. Notably, MMP7 is a powerful enzyme, which in addition to Fn aggregates, also cleaves other ECM proteins, as well as cell surface receptors and growth factors [215]. This emphasizes the importance of local, targeted, and controlled MMP7 delivery to Fn aggregates.

Additionally, Fn aggregation may be influenced by heat shock proteins (HSPs). HSPs are intracellular chaperones that aid in the folding of denatured proteins [216]. Their extracellular presence increases in response to injury and under stress [217,218]. Proteomic analysis indicates that Fn aggregates serve as a scaffold for HSP70, which in turn induce both pro- and anti-inflammatory phenotypes in microglia and macrophages [184]. Furthermore, HSP70 increases the expression of ECM proteins, such as collagen I and Fn via transforming growth factor type β1 (TGF-β1) [219]. An exacerbated increase in HSP70 expression in response to heat shock and LPS stimulation was found in immune cells from MS patients compared with healthy subjects [220]. Furthermore, HSP47 and HSP90β are associated with Fn aggregates [184]. While HSP47 is involved in fibrillar collagen deposition [221,222], HSP90β contributes to the unfolding of Fn dimers to facilitate Fn fibrillogenesis [223]. Notably, the presence of antibodies against HSP90β is elevated in CSF of MS patients and implicated in OPC death [224], indicating a role for HSP90β in MS pathogenesis. These data point to the idea that, in MS lesions, HSP dysfunction may contribute to Fn aggregation and that the accumulation of HSPs in Fn aggregates impairs the functioning of glial cells.

**Table 1:** Potential therapeutic strategies to overcome the fibronectin-aggregate-mediated inhibition of remyelination failure.

Strategy	Method	Mechanism of Action	Reference
Prevent Fn expression	Prevent TG2 expression or activity	Mediates Fn expression and deposition	[161,225]
Prevent Fn aggregation	Modulate Fn splicing	Induces conformational changes in Fn to increase cell surface binding	[198]
Prevent Fn aggregation	Prevent TLR3 signaling (astrocytes)	Prevents the release of Fn fibrils from the cell surface	[198]
Prevent Fn aggregation	Modulate HSP90β activity	Contributes to the unfolding of Fn to facilitate Fn fibrillogenesis	[223]
Degrade Fn aggregates	Increase MMP7 expression and activity	Cleaves Fn, including Fn aggregates	[211]
Bypass Fn aggregates	Treat with ganglioside GD1a	Overcomes the Fn-mediated inhibition of OPC maturation via a PKA-mediated signaling pathway	[18]
Bypass Fn aggregates	Treat with PDE inhibitors	Prolongs cAMP levels, thereby potentially activating PKA, and enhances CNS remyelination	[194,195,196,197]

cAMP—cyclic adenosine monophosphate; CNS—central nervous system; Fn—fibronectin; HSP90β—heat shock protein 90 beta; MMP7—matrix metalloproteinase 7; PDE—phosphodiesterase; PKA—protein kinase A; TG2—tissue transglutaminase 2; TLR3—Toll-like receptor 3.

In conclusion, promising options to overcome the impairment of remyelination by Fn aggregates are (1) bypassing its signals to OLG lineage cells by GD1a, (2) preventing its aggregation by interfering with TLR3 signaling and/or HSP function, and (3) facilitating its clearance via the lesional delivery of MMPs, such as MMP7 (Table 1). Via stereotactic intralesional injection, the pharmacological effect of GD1a was documented [18], while the efficacy of the other potential therapeutic strategies still requires testing in relevant experimental models. Notably, as in the absence of astrocytic or plasma Fn, remyelination still occurs [74] and the transient increase in Fn upon toxin-induced demyelination is redundant for remyelination, making the degradation of its aggregates or even premature degradation of Fn feasible approaches to overcome remyelination failure in MS. For the latter, downregulation of tissue transglutaminase 2 (TG2) is an attractive option, as astrocytic TG2 mediates Fn expression and deposition [161,225].

Nevertheless, the beneficial effects of Fn should be considered when designing therapeutic strategies. In particular, Fn’s involvement in BBB maintenance complicates targeting Fn for myelin regeneration in MS, as altering the functioning or presence of this protein may adversely affect the BBB. For example, MMP7 may negatively affect BBB functioning by promoting the breakdown of the BBB when administered peripherally. Indeed, in patients suffering from traumatic brain injury serum levels of MMP7 correlated with dynamic contrast-enhanced magnetic resonance imaging (DCE-MRI), which measures the BBB permeability and breakdown [226]. Furthermore, elevated MMP9 serum levels were observed in RRMS patients, with higher serum levels predicting new gadolinium-positive lesions, i.e., active lesions with a disrupted BBB [227]. Targeting Fn aggregates directly by increasing MMP activity may therefore not be the most suitable tactic for preventing the remyelination block observed in MS, especially when these changes are effectuated on a systemic rather than local level. Moreover, despite BBB alterations in the early stages of MS, PPMS is characterized by lesions with a different inflammatory profile, where the BBB remains largely intact and remyelination is marginal [228]. This highlights the necessity for a ‘two-step approach’, i.e., brain-targeted and locally delivered therapeutics. Nevertheless, this is complicated when treating brain diseases [229,230], mainly due to the presence of the BBB, for which solutions will be discussed next.

## 6. MS Therapeutics: Drug Delivery Vehicles for Delivery to the Brain

Plasma proteins and other compounds that are neurotoxic at high concentrations damage neurons and other brain-resident cells when allowed free access to the brain. For example, the blood plasma components thrombin and plasmin can induce apoptosis or lead to seizures [231,232], while high levels of the excitatory neurotransmitter glutamate are toxic to neurons [233]. Therefore, passive diffusion of hydrophilic and hydrophobic compounds across the BBB is restricted by the presence of TJs and efflux transporters, respectively, while active transport through substrate-specific transporters and receptor-mediated transcytosis allows for the regulated uptake and excretion of compounds [234] in order to maintain brain homeostasis (Figure 1).

The encapsulation of drugs in a carrier system that is able to cross the BBB seems a promising strategy for obtaining brain penetration of medicinal compounds while providing several additional advantages [235]. Techniques are being used to develop nanocarriers with a high drug-loading profile [236] and those that aid controlled and sustained release of the drug of interest [237,238,239,240]. By doing so, the need for frequent dosing is reduced. Nanovehicles can improve the bio-availability of hydrophobic and hydrophilic compounds by providing protection against chemical and biological degradation and improving target-site delivery [241,242,243]. Additionally, nanoparticle design can be optimized for the development of precision medicine, where the personal characteristics of patients in conjunction with specified nanomedicine engineering allow for patient-specific disease treatments [244].

However, systemically delivered nanocarriers still face several hurdles, including the acidic environment of the gastrointestinal tract (when administered orally), clearance by the liver and spleen, clearance by immune cells, and physical barriers that prevent easy access to target sites [245]. Overall, for in vivo efficacy, nanocarriers need to show high stability, low toxicity, prevent clearance by the reticuloendothelial system, and efficiently accumulate at the target site [229,246,247]. Hence, the physicochemical properties of the nanocarrier (i.e., size, shape, charge, and type of material) and the properties of the biological barriers that hinder their transport to the target site need to be taken into account [245,248], as well as how these properties influence the interaction between the nanocarriers and cell barriers. Furthermore, a myriad of nanocarrier modifications are invented, which are aimed at the sustained release of the drug, target specificity, and circumvention of intra- and extracellular clearance. For example, tuning the charge of lipid nanoparticles (LNPs) resulted in tissue-specific gene delivery by LNPs [249]. Furthermore, analyte-responsive hydrogels bind or release drugs of interest in a controlled manner, i.e., glucose oxidase-containing hydrogels can interact with glucose in the environment, swell, and subsequently release insulin [250,251,252]. Moreover, reversible PEGylation enhances the stability and circulation time of nanocarriers in vivo without preventing target cell uptake and drug release [253].

Commonly studied nanocarriers are lipid- or polymer-based and include liposomes, polymersomes, micelles, dendrimers, nanogels, nano-emulsions, and exosomes [229,254,255]. Unfortunately for brain-targeted therapeutics, the brain endothelium (BBB) appears more difficult for nanoparticles to penetrate than lung, liver, and kidney endothelial barriers [256]. Several studies utilized nanocarrier formulations with either the aim to improve the delivery of established drugs that reduce the number and severity of relapses in MS or to test experimental therapeutic agents in experimental models of MS aimed at alleviating disease progression (Table 2) [257,258,259,260,261,262,263,264,265,266,267,268,269,270,271,272,273,274,275,276,277,278,279].

### 6.1. Lipid-Based Nanoparticles as Drug Delivery Vehicle for RRMS Treatment

**Figure 3:**
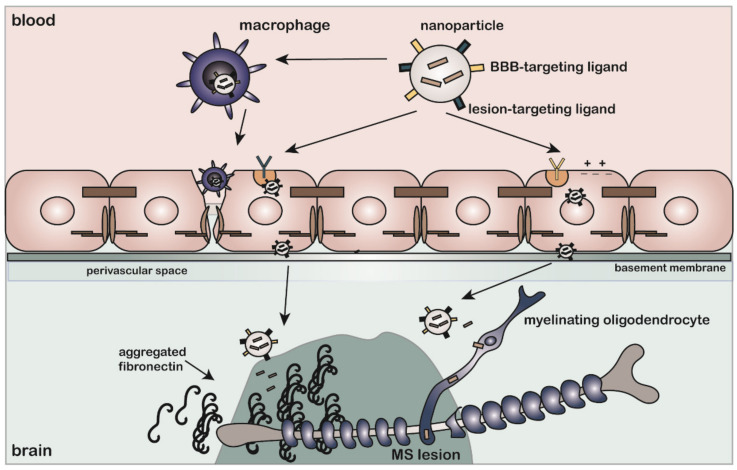
Strategies to deliver MS therapeutics that overcome the fibronectin-mediated inhibition of remyelination failure in the brain. Therapeutic compounds encapsulated in nanoparticles that contain a blood–brain barrier (BBB)-targeting ligand or carry a positive charge enter the brain via transcytosis through receptor-mediated transcytosis (RMT) or adsorptive-mediated transcytosis (AMT), respectively. Alternatively, in relapsing–remitting MS, surveilling monocytes may phagocytose nanoparticles and transport these across the compromised BBB during relapses. Furthermore, the identification of lesion-specific BBB alterations (i.e., upregulation of receptors at the BBB near lesions) would aid the targeting of lesion-directed medication. In the brain, lesion targeting of therapeutic-containing nanoparticles may be achieved by cell-specific ligands targeting receptors that are present on, e.g., oligodendrocyte lineage cells, or ligands targeting the altered, and therefore specific, environment in MS lesions. For example, targeting specific splice variants of fibronectin that are abundant in fibronectin aggregates can aid the cell- and lesion-specific delivery of the therapeutic compound. This ‘two-step approach’ utilizes ligands that facilitate transcytosis across brain endothelium (BBB) and ligands that direct the delivery of therapeutics to MS lesions.

Liposomes are small biocompatible vesicles that consist of one or multiple lipid bilayers. Commonly used lipids include cholesterol, phosphatidylcholine, and soy lecithin [280]. Liposomes can entrap hydrophilic and lipophilic compounds in their aqueous core and lipid bilayer(s), respectively [229,281], thereby making liposomes versatile nanocarriers for a multitude of therapeutics. The addition of PEG moieties to the outer surface of liposomes endows them with stealth properties, limiting an immune response and reducing their plasma clearance [282]. The biodistribution and brain penetration of liposomes were tested in relevant experimental models for MS. The temporary BBB disturbance observed in RRMS is equally observed in experimental MS models that simulated its inflammatory profile, such as EAE. This allowed researchers to test liposomal treatments for MS that are not specifically targeted to the BBB [257]. For example, in EAE, ^99m^Tc-DTPA-labeled liposomes accumulated more in the brain and spinal cord compared with healthy controls, which is an effect that may rely on BBB disruption and/or infiltrating macrophages that transport liposomes to the CNS (Figure 3) [258]. Notably, DTPA is a low-molecular-weight molecule that can cross the BBB during injury or inflammation-induced endothelial permeability. Compared with free DTPA, DTPA conjugated to liposomes accumulated more in lesioned areas, which is an effect that could be attributed to monocytes that phagocytosed liposomes before significant BBB damage had occurred (Figure 3) [257]. In addition to employing infiltrating liposome-laden monocytes as a means of transporting anti-inflammatory drugs to target inflammatory lesions in MS [259], Schweingruber et al., provided evidence that glucocorticoids, which are approved MS therapeutics, entrapped in liposomes induced macrophages to adopt an anti-inflammatory phenotype rather than inducing T cell apoptosis [262]. Similarly, glucocorticoids entrapped in inorganic–organic hybrid nanoparticles (IOH-NP) were shown to exclusively modulate macrophage functioning [283]. Conversely, using a long-circulating prednisolone liposome formulation, Schmidt et al., demonstrated that glucocorticoid administration via liposomes restored BBB integrity, reduced inflammation caused by T cells, diminished macrophage infiltration into the CNS, and slowed down disease progression in EAE [261]. Active targeting of glucocorticoid liposomes to the brain was also achieved by labeling liposomes with glutathione, which is a BBB-targeting ligand [264].

Another approved MS medication is dimethyl fumarate (DMF), which is an orally administered anti-inflammatory drug with neuroprotective properties [284,285]. Due to its low brain permeability and oral administration, it requires high and frequent dosing. Accordingly, the drug may benefit from incorporation into a nanocarrier system. Indeed, the encapsulation of DMF into solid lipid nanoparticles increased the half-life and bioavailability of the orally administered drug in rats [265]. In the cuprizone model, DMF-loaded lipid-based nanoparticles administered orally once a day improved remyelination to the same extent as a three-times a day free oral DMF treatment [266]. Hence, incorporation into nanoparticles reduces the need for excessive dosing, particularly for a drug with a fast clearance rate. Other than the encapsulation of approved MS medication in liposomes, the encapsulation of autoantigens [267] and MBP-peptides [275,276,278,286] in liposomes was successfully tested as a potential disease-modifying medication. Thus, there is compelling evidence that the encapsulation of approved or experimental therapeutics in liposomes allows for the safe administration of drugs in MS patients that cannot be administered in free-form due to their instability in circulation and/or can have beneficial effects on top of the curative response of the drug itself, such as less frequent dosing.

**Table 2:** Nanoparticles used for treatment in experimental MS models and in MS patients.

Treatment	Administration Means	Administration Time Point	Outcome Measure	Reference
**Experimental MS Models**				
^99m^Tc-DTPA-loaded liposomes in EAE	Intravenous	At induction of disease	Biodistribution of liposomes	[258]
MOG_40–55_-loaded liposomes in EAE	Intraperitoneal	At induction of disease	Preventive and preclinical treatment effects on EAE development	[267]
MBP-loaded liposomes in EAE	Subcutaneous	At disease onset for 6 days	Effect of different MBP isoforms on EAE progression	[275]
Prednisolone-loaded liposomes in EAE	Intravenous	At peak of disease	Effect on EAE progression, BBB permeability, and drug biodistribution	[261]
(Methyl)prednisolone-loaded liposomes in EAE	Intravenous	At peak of disease	Effect on EAE progression and macrophage functioning	[262]
Methylprednisolone-loaded liposomes in EAE	Intravenous	Prophylactic, at disease onset, and disease peak	Brain-targeted effect on EAE symptoms	[264]
MOG-loaded PLGA particles in EAE	Intravenous/subcutaneous	Prophylactic	Effect on EAE development	[268]
MOG-anti-Fas-PD-L1-Fc-CD47-Fc-TGFβ-loaded PLGA particles in EAE	Intravenous	At disease onset and disease peak	Modulation of auto-reactive T cells in EAE and disease progression	[269]
MOG-IL10-loaded PLGA particles in EAE	Subcutaneous	Prophylactic, at disease onset, and disease peak	Effect of ‘inverse vaccination’ on EAE progression	[270]
PLP-coupled PLGA particles in EAE	Intravenous	At disease onset	Treatment of EAE and nanoparticle uptake in vitro by antigen-presenting cells	[272]
PHCCC-loaded PLGA particles in EAE	Subcutaneous	From induction of disease, every 3 or 5 days	Effect on DC activation and EAE disease progression	[273]
miR-219a-5p liposomes, PLGA particles, and extracellular vesicles in EAE	Intranasal	2 and 8 days post-induction of disease (before symptom onset)	Effect on remyelination in EAE	[274]
Curcumin-loaded HPPS in EAE	Intravenous	8, 10, 12, and 14 days post-induction of disease	Restriction of immune cell infiltration of the brain in EAE by modulation of monocytes	[259]
PLP-coupled PLGA particles in relapsing–remitting EAE	Intravenous	At disease onset, disease peak, and disease remission	Prevention and treatment of relapsing EAE disease	[271]
(Methyl)prednisolone-loaded liposomes in chronic relapsing EAE	Intravenous	At first peak of disease	Effect on disease progression, their effect on relapse risk, and macrophage CNS infiltration	[263]
Dimethyl-fumarate-loaded solid lipid nanoparticles in cuprizone	Oral	Daily cuprizone and nanoparticles for 30 days	Effect on remyelination	[266]
LIF-loaded PLGA particles in focal demyelination	Intralesional	8 days post-lysolecithin lesioning	Effect on OPC differentiation in vitro and remyelination in vivo	[279]
**MS Patients**				
MBP-loaded liposomes	Subcutaneous	Once a week for 6 weeks	Safety profile of CD206-targeted liposomal MBP treatment in RRMS and SPMS patients	[286]
MBP-loaded liposomes	Subcutaneous	Once a week for 6 weeks	Serum cytokine analysis and Th1/Th2 ratio in RRMS and SPMS patients	[278]

BBB—blood–brain barrier; CNS—central nervous system; DTPA—diethylenetriaminepentacetate; EAE—experimental autoimmune encephalomyelitis; HPPS—high-density lipoprotein-mimicking peptide-phospholipid scaffold; LIF—leukemia inhibitory factor; MOG—myelin oligodendrocyte glycoprotein; MS—multiple sclerosis; OPC—oligodendrocyte progenitor cell; PHCC—N-phenyl-7-(hydroxyimino) cyclopropa[b]chromen-1a-carboxamide; PLGA—poly(lactic-co-glycolic acid); RRMS—relapsing–remitting MS; SPMS—secondary progressive MS.

### 6.2. Polymer-Based Nanoparticles as a Drug Delivery Vehicle for RRMS Treatment

Alongside lipid-based nanoparticles, polymeric nanoparticles were demonstrated to be an effective and safe drug delivery system, though for clinical applications, the toxicity profile of polymeric particles needs to be critically assessed. Poly(lactic-co-glycolic acid) (PLGA) is a synthetic polymer that shows potential for clinical applications due to its biocompatibility, biodegradability, and low immunogenicity profile. Despite its promise, few PLGA-based medications are currently approved for clinical use [287]. Variations in product design may underlie the low success rate of PLGA nanospheres on the market. Since minor alterations in the production process may alter the pharmacodynamics of the desired product, a strong emphasis needs to be placed on the optimization of particle properties [288]. PLGA particles consist of polymerized lactic acid and glycolic acid subunits. The proportion of lactic acid to glycolic acid determines the hydrophobicity of the particle, with higher proportions of lactic acid conferring a higher degree of hydrophobicity. Simultaneously, lactic-acid-rich PLGA particles degrade slower than particles relatively high in glycolic acid, though particles with equal amounts of lactic acid and glycolic acids degrade the fastest [289,290]. The therapeutic effectiveness of PLGA particles depends on their physicochemical properties, drug loading efficiency, and drug release behavior, which altogether determine successful drug delivery. Biodistribution and (immune) clearance of PLGA nanoparticles are largely determined by their size and surface charge (zeta potential) [291]. Properly designed PLGA particles demonstrated improved delivery of therapeutics in vivo. For example, the incorporation of the chemotherapeutic docetaxel in pegylated PLGA particles showed minimal liver accumulation in rats, enhanced accumulation in tumors in mice, and induced tumor shrinkage in humans at a lower dose than when free docetaxel was administered [292].

PLGA particles are tested as vehicles to induce immune tolerance in experimental models of MS. Inhibition of the inflammatory phenotype of autoreactive T cells and a delay in disease onset were achieved by injecting PLGA particles containing myelin oligodendrocyte glycoprotein (MOG) prior to the induction of EAE [268]. Similarly, an ‘inverse vaccination’ treatment with MOG-PLGA, PLP-PLGA, and IL10-PLGA particles inhibited EAE development and equally ameliorated EAE progression when administered post-EAE induction [270,271]. In vitro data suggest that disease-relevant peptide-conjugated PLGA nanoparticles diminished inflammatory signaling in macrophages and dendritic cells, i.e., antigen-presenting cell types with a known role in nanoparticle clearance from blood circulation. These in turn reduced T cell proliferation and induced T cell apoptosis [272]. Other compounds (indirectly) affecting T cell polarization also benefit from encapsulation in nanoparticles. For example, (hydroxyimino)cyclopropa[b]chromen-1a-carboxamide (PHCCC) affected glutamate metabolism in dendritic cells, which indirectly affected T cell polarization through cytokine secretion. The incorporation of PHCCC in PLGA particles resulted in a controlled release of PHCCC, thereby allowing for a reduction in dosing frequency from daily to once every three days in mice [273].

### 6.3. Drug Delivery Vehicles for Treatment of Progressive MS

To date, most approved MS drugs modulate the peripheral immune system with the purpose of reducing the inflammatory response associated with relapses [293]. Hence, most previously described studies targeting MS rely on the presence of a disrupted BBB or a significant inflammatory response characterized by infiltrating macrophages. As stated earlier, treatment aimed at restoring remyelination in progressive MS when inflammation has largely subsided requires BBB-penetrating capabilities of drugs or nanocarriers that ideally recognize chronic lesions (i.e., with a low inflammatory profile) and/or target cells within the lesion. Few nanoparticle studies have specifically targeted OLGs in MS or assessed nanoparticle accumulation in the brain when the BBB is largely intact to determine whether particles could cross the BBB. A recently published study by Osorio-Querejeta et al., compared liposomes, PLGA particles, and extracellular vesicles (exosomes) for the delivery of miR-219a-5p, which is a microRNA capable of inducing OPC differentiation and myelination [274]. Liposomes and PLGA particles were more efficiently taken up by OPCs in vitro, though exosomes were more effective at inducing OPC differentiation, as assessed by the expression of myelin-related genes. Additionally, in an in vitro BBB model, exosomes crossed the BBB more easily than liposomes or PLGA particles. Intranasal delivery of miR-219a-5p-containing exosomes after EAE induction enhanced remyelination and attenuated clinical disability scores compared with treatment with control exosomes. This study demonstrated the effectiveness of remyelination-inducing therapy, albeit in conditions where inflammation was present. As inflammation-induced alterations in the vasculature are inherent to EAE, it is likely that particles could reach the brain through a breached BBB. Indeed, the binding of cationic liposomes to endoneural vessels in the spinal cord occurred throughout the disease course of EAE, which was not observed for control animals. This binding effect was correlated to changes in animal’s vasculature and inflammatory profile [294]. For this reason, testing remyelination therapy in non- or low-inflammatory conditions is necessary to understand the applicability of these therapies to progressive MS.

Leukaemia inhibitory factor (LIF) is a known pro-myelinating factor shown to improve remyelination [295,296,297]. A single treatment with LIF-containing PLGA particles induced differentiation of OPCs into mature OLGs by activating pSTAT-3 signaling in vitro. In vivo, these particles increased myelin thickness, as well as the percentage of remyelinated axons after a focal demyelinating insult [279]. OPC-specific targeting was achieved by decorating nanoparticles using anti-NG2 antibodies, thereby avoiding off-target effects. As LIF is rapidly degraded in vivo, the utilization of PLGA particles improved the stability of the drug [279]. However, as particles were injected directly into the demyelinated lesions, systemic stability and the BBB-traversing capability of the particles were not assessed. Hence, an effective drug delivery system for the treatment of progressive MS that is systemically administered and targeted to the brain needs to be developed, for which considerations and potential strategies are reflected upon in the next section.

## 7. Progressive MS Treatment: Considerations for Designing a Brain-Targeted Drug Delivery System

The ideal drug delivery system for the treatment of progressive MS shows no toxicity, has high specificity for the target site (i.e., the brain and specifically demyelinated lesions), only releases the drug when it has arrived at the lesioned area, and is biodegradable and/or biocompatible. To achieve such a highly specific delivery of the therapeutic compound, several complicating factors must be addressed. Although endocytosis followed by intracellular disintegration of the nanoparticle and subsequent drug release is required for the drug to reach its intracellular targets, first, transcytosis across the endothelial cells of the BBB is needed to get from the blood to the brain. Several strategies for blood-to-brain transport are developed, as well as alternatives to circumvent the BBB, which are discussed next in more detail.

### 7.1. Receptor-Mediated Transcytosis (RMT)

A commonly used brain-targeting approach uses the conjugation of BBB endothelial cell-recognizing ligands, targeting peptides, or antibodies to nanoparticle formulations, which allows them to cross the BBB. Examples include nanoparticles decorated with ligands for the transferrin (Tf), insulin, lipoprotein (LRP), lactoferrin (LfR), and diphtheria toxin receptors; the GM1-binding G23 peptide; and glutathione [264,298,299,300,301,302,303,304,305,306,307,308]. Many of these brain-targeting peptides and ligands cross the BBB through ATP-dependent receptor-mediated transcytosis (RMT) [309]. RMT is the process by which ligand binding to membrane-bound receptors induces internalization of the ligand–cargo complex through endocytosis, followed by intracellular vesicular trafficking and exocytosis of the cargo at the opposite side of the endothelial cell monolayer (Figure 1) [114]. The use of specific coatings and nanoparticle materials may hence prove to be important for organ-targeted drug design [256]. For example, a poloxamer-188 coating caused the adsorption of blood apolipoproteins to the surface of PLGA particles, which then induced BBB transcytosis through an interaction with the LDL receptor (LRP1) [310]. Poloxamer-188-coated PLGA particles demonstrated efficient BBB transcytosis in vitro and successful delivery of an anti-viral HIV drug to macrophages and microglia [311,312].

### 7.2. Adsorptive-Mediated Transport (AMT)

Another strategy to target the brain is the utilization of positively charged moieties that mimic the transport of polycationic proteins, such as protamine, across the BBB [313]. The use of cationic polymers promotes BBB transport through adsorptive-mediated transport (AMT) [313,314]. AMT relies on electrostatic interactions between the negatively charged endothelial cell membrane and the positively charged molecule (Figure 1). Based on the same electrostatic interactions, the cationic polymer poly(β-amino ester) (PbAE), when mixed with siRNA, self-assembled into 100 nm sized nanoparticles and released the siRNA content when exposed to the reducing environment of the cytosol [315]. In an iPSC-derived human BBB transwell model co-cultured with glioblastoma cells, the PbAE particles delivered siRNA to glioblastoma cells after transcytosis by the in vitro BBB. Successive in vivo experiments demonstrated that these particles reached and delivered siRNA to orthotopically implanted patient-derived glioblastoma cells in mice after intravenous administration [316].

Some cell-penetrating peptides (CPPs), such as HIV-1 trans-activating protein (TAT), also employ AMT [317]. The decoration of nanoparticles with CPPs strongly increased transcytosis and improved targeted brain delivery and controlled release of nanoparticle contents [318,319,320]. Liposomes decorated with a combination of the cyclic Arg-Gly-Asp (cRGD) peptide, which binds to integrin αvβ3 at the BBB [321] and induces clathrin-mediated endocytosis in cells [322], with a histidine-rich pH-sensitive cell-penetrating peptide (TH) that evades lysosomal degradation [323] resulted in their efficient transcytosis across the BBB endothelium. After binding to integrin αvβ3, nanoparticles were internalized by glioma cells due to the positive charge of the CPP, which was achieved via histidine protonation in the acidic microenvironment around the tumor cells [318].

### 7.3. Focused Ultrasound

Besides ligand-based nanoparticle modifications aimed at transporting drugs across tightly connected BBB endothelial cells, temporarily opening the BBB through focused ultrasound (FUS) is also considered a viable method of brain-targeted drug delivery [324]. The technique, which was first described approximately twenty years ago [325,326], involves the local application of pulsed sonication. In combination with gas-filled microbubbles, reversible openings in the BBB can be achieved, through which therapeutics gain access to the brain [324]. Since then, FUS has demonstrated promising benefits for the treatment of neurodegenerative diseases [327,328,329,330]. FUS appears to rely on both paracellular and transcellular transport mechanisms. Thus, FUS induces the temporary disintegration of TJ complexes, thereby allowing for paracellular entry into the brain [331], and was also shown to increase endocytosis [332,333]. With the appropriate ultrasound settings, it has promise for the selective delivery of medication into the brain, though its safety and application in humans still need to be properly assessed. For application in MS, it is disadvantageous that individual lesions need to be targeted, which is complex when using FUS.

### 7.4. Intranasal Drug Delivery

A way to circumvent the BBB is by administering drugs intranasally. A portion of intranasally administered particles is expected to reach the brain via trigeminal neurons and olfactory nerves without entering the systemic circulation. The CSF can be reached directly via a route that involves the nasal epithelium and the perineuronal and subarachnoid space [334,335]. Direct nose-to-brain delivery through the olfactory bulb may involve paracellular, transcellular, and neuronal transport [335]. Thus, intranasal dispensation of nanoparticles, e.g., PLGA particles [336,337,338], and liposomes [339,340,341] may offer a means of fast and efficient delivery of brain therapeutics. However, as the nasal cavity is small, only a limited amount of the drug can be administered at each dose. Furthermore, mucociliary clearance and enzymatic degradation in the nasal cavity reduce brain uptake of the administered drug [342], causing less than 1% of the drug administered to reach the brain [343]. For this reason, enzyme inhibitors, mucoadhesives, and absorption enhancers are incorporated in intranasal formulations, which themselves can be irritating to nasal mucosa [343,344]. In addition, the efficiency of intranasal drug delivery across olfactory cells in vitro differed between PLGA and lipid carriers (with lipid carriers having a higher transcytotic ability) [320]. Furthermore, though intranasal drug delivery is promising, it appears to not evade systemic circulation completely [345]. Thus, BBB targeting through RMT or AMT may still be relevant to nanoparticles following nasal administration.

## 8. MS Therapeutics: Considerations for Intracellular Delivery of Therapeutic Agents

An important consideration for nanocarrier design for the treatment of MS are properties that allow systemically administered nanoparticles to release their content only after crossing the BBB and accumulation at the target site, i.e., an MS lesion (Figure 3). Upon endocytosis of nanoparticles by target cells, the therapeutic payload needs to escape from the endolysosomal system to circumvent the acidic and enzymatic environment of the lysosome that may destabilize or inactivate therapeutics [346,347,348]. Where hydrophobic drugs can passively cross the endosomal membrane, intracellular delivery of hydrophilic drugs, e.g., DNA, RNA, and proteins, requires permeabilization of the endosomal membrane [349,350,351]. At least for mesoporous silica nanoparticles, repeated administration resulted in reduced intracellular delivery of hydrophilic cargo [346], which signifies that the development of nanocarriers with a high drug-loading capacity is important. PEGylation, which is commonly used to confer stealth properties on nanoparticles to promote their blood circulation time, has an inhibiting effect on endo/lysosomal escape. The addition of PEG lipids onto gene-carrying liposomes inhibited the endosomal release of the genetic material and thus gene delivery [352,353,354]. To overcome this limitation, exchangeable [354], cleavable [355,356], and pH-sensitive PEG chains are used [357]. Finally, small extracellular vesicles (sEVs) were shown to enter cells via endocytosis and fuse with endosomal and/or lysosomal membranes in order to release their cargo in the cytosol [358]. As it was demonstrated that sEVs could efficiently cross the BBB and deliver a pro-myelinating drug in vivo [274], their applicability for brain-targeted medicinal delivery seems promising. Overall, nanoparticle design aims for nanoparticle stability during systemic circulation and the release of therapeutics, i.e., nanoparticle destabilization, at the target site.

## 9. Active Targeting to MS Lesions: Considerations for Controlled Drug Delivery to Overcome Fibronectin-Mediated Inhibition of Remyelination

While the previous sections largely focused on drug vehicles’ requirement to cross the BBB, drug delivery to demyelinated lesions also requires lesion-targeting approaches. To this end, conjugation of nanoparticles with ligands, such as antibodies or peptides that interact with binding sites on lesion-resident cells or environmental factors that are only present in MS lesions, ensures controlled drug delivery. In particular, peptides are favorable for being small, easy to synthesize, and less immunogenic than antibodies. In the following, we reflect on how to control and functionally deliver medication to MS lesions to overcome the Fn-mediated inhibition of remyelination failure. The need for nanoparticles targeting Fn aggregate-bearing lesions is essential for drugs that may interfere with Fn expression and/or degradation, as these may also affect Fn in the BBB BM without lesion-specific targeting. Contrariwise, drugs that interfere with Fn aggregation or bypass the negative effect of Fn aggregates on OPC maturation and have minimal off-target effects are unlikely to interfere with Fn functioning outside of lesions. Additionally, similar delivery approaches can be considered for other remyelination-directed medications. For an overview of peptide-based targeted drug delivery to cells and ECM components in MS lesions, we refer to a recent comprehensive review [359].

### 9.1. Active Targeting to Fn Aggregates

Active targeting of Fn aggregates is relevant for both functional cellular delivery of therapeutic agents that bypass Fn-mediated inhibition of remyelination (e.g., GD1a), and treatment approaches aimed to clear Fn aggregates at MS lesions (e.g., MMP7), with the latter requiring extracellular release of nanoparticle content or local stimulation of MMP production. As mentioned before, Fn is not abundantly present in healthy adult brains, allowing for Fn-targeting approaches in demyelinated areas. In addition, the targeting of Fn was utilized for drug delivery to tumor tissues [360,361,362,363]. The EDA and EDB splice variants of cellular Fn are mainly expressed during fetal development but become re-expressed at locations of tumor growth [360]. Using phage display, a peptide recognizing the EDB splice variant of Fn was identified that showed specific targeting to human prostate tumor xenografts implanted in mice [364]. Moreover, imaging of xenografted tumor tissue in mice was achieved using an EDB Fn-splice-variant-binding high-affinity peptide (named an aptide) [365]. Conjugation of these aptides to liposomes or PLGA particles [366,367,368] and an aptide–docetaxel conjugate [369] improved chemotherapy delivery to the tumor and inhibited tumor growth (reviewed in [359]). In addition, liposomes decorated with an aptide recognizing the EDB domain of Fn showed improved drug delivery to MCF7/ADR orthotopic tumors in vivo and delayed tumor growth [363]. One of the features of Fn aggregates in demyelinated MS lesions is their relative abundance in the EDA over the EDB splice variant [74,198]. This hints at the possibility of using an EDA-recognizing ligand to target Fn aggregates in MS. Advantageously, this method of interaction with plasma Fn can be circumvented, as EDA is unique to cellular Fn. Future studies need to uncover whether such Fn-targeted nanoparticles will indeed reach MS lesions. A complicating factor is that in healthy brain tissue, EDA Fn in the basement membrane is limited to larger blood vessels, while in actively demyelinating MS lesions, EDA Fn is abundantly present in perivascular networks [107]. It would be undesirable if nanoparticles that targeted lesional Fn aggregates remain within the perivascular space rather than penetrate the lesioned parenchyma. However, this may not be a problem in chronic demyelinated MS lesions, as in chronic MS lesions, EDA Fn is less abundant in the basement membrane, while parenchymal Fn aggregates are more abundant [17] and remyelination failure is more prominent [14,17]. Alternatively, other ECM components in MS lesions can be utilized as bait for peptide-targeted drug accumulation in MS lesions [359]. For example, a CSPG-targeting peptide was used for functionalizing nanoparticles to target traumatic brain injury [370].

### 9.2. Active Targeting of Cells

Alternatively, functional delivery of ganglioside GD1a to OLG lineage cells, which has minimal off-target activity in healthy tissue, and specificity to overcome Fn-induced remyelination failure can be employed [18]. The use of a nanocarrier conjugated with OLG lineage cell-targeting peptides or antibodies may facilitate specific delivery to the cells of interest. To this end, anti-NG2-antibody-coated nanoparticles were successfully used to functionally deliver LIF to OPCs [279], while targeting OPCs with anti-PDGFRα antibodies offers an alternative approach [371]. Given that GD1a is hardly degraded in OLG lineage cells and the addition of GD1a to OPCs can overcome Fn-mediated inhibition of myelin membrane formation in vitro [18], GD1a incorporation in anti-NG2 or anti-PDGFRα antibody-coated nanoparticles may be a feasible approach. As an alternative, nanocarriers decorated with antibodies against GPR17 may be considered. GPR17 surface expression is restricted to immature OLGs and is absent on mature myelinating OLGs [372]. Strategies to prevent Fn synthesis (e.g., with the enzyme TG2) and/or to interfere with aggregation (e.g., with TLR3 antagonists) may benefit from functional delivery of therapeutic cargo to astrocytes present in MS lesions. Astrocyte-targeting peptides were identified [373,374,375]; however, given astrocyte heterogeneity per se and functional remodeling of astrocytes in response to demyelination and inflammation, the knowledge on selective targeting of astrocyte subpopulations, and therefore, the suitability of these peptides to specifically target astrocytes in MS lesions, is still limited. Nevertheless, to specifically target cells in MS lesions, exploiting a peptide that binds to a lesion-specific environmental factor, e.g., Fn or other ECM components that are upregulated in MS lesions, likely represents an effective approach to accomplishing lesion-specific cellular drug delivery method for lesion-targeted functional drug delivery [359].

## 10. Concluding Remarks and Future Perspectives

MS is a heterogeneous disease involving inflammation, as well as neurodegenerative processes that simultaneously contribute to disease pathology. The underlying cause of MS is still unknown and likely to differ between patients. This heterogeneity in disease pathology asks for a multitude of treatment approaches as each stage of the disease and likely each lesion type has its hallmarks. Therefore, no single MS treatment is appropriate for all MS patients, nor will it be effective for all the different disease stages. This means that MS treatment is likely to benefit from personalized medicine. During later stages of the disease, impaired remyelination results in secondary neurodegeneration that overshadows the initial flares of demyelinating insults, meaning that a sustained and gradual decline in neurological functioning appears. Current therapeutic approaches mainly focus on mitigating the inflammatory components of the disease but do not halt underlying degenerative processes affecting disease progression. Several factors that are addressed in this review, including changes in ECM composition and stiffness, contribute to remyelination failure. Moreover, BBB malfunctioning appears to underlie disease initiation, while a relatively intact BBB concomitantly impedes disease treatment in progressive MS. We focused on a particular ECM aberration that is typical of chronically demyelinated lesions, namely, the occurrence of remyelination-impairing Fn aggregates, and discussed distinct means to specifically negate the effect of Fn aggregates. Given the role of Fn in BBB maintenance and angiogenesis, the release of pro-remyelination drugs that interfere with Fn signaling at the BBB should be prevented. Therefore, to overcome Fn-mediated inhibition of remyelination in chronic demyelinated lesions, we stress the importance of employing nanocarrier systems with a ‘two-step approach’: decoration of drug-containing nanoparticles with ligands that facilitate transcytosis across the BBB and delivery to Fn aggregates, OPCs, or astrocytes for lesion-specific drug release. Ideally, these nanocarriers possess a high drug-loading capacity, shield the drug from environmental influences, allow for controlled release of the drug, decrease the need for frequent dosing, and reduce undesirable side effects. Lipid (liposomal)- and polymer (PLGA)-based nanomedicines are being used for (experimental) MS nanomedication. Whether one or the other would be most suitable for remyelination-based treatment of MS lesions depends on the type of therapeutic to be delivered. For instance, hydrophilic and amphiphilic drugs have a low encapsulation efficiency into PLGA particles in contrast to hydrophobic compounds [376]. In turn, liposomes can encapsulate hydrophilic compounds in their aqueous core, preventing rapid clearance and enabling sustained release [377], and amphiphilic molecules in their lipid bilayer. For example, to bypass Fn aggregate-mediated inhibition of remyelination, the encapsulation of ganglioside GD1a, which is an amphiphilic compound, into liposomes would be preferred. Notably, lesion-specific alterations of the BBB and its BM were reported in MS [378], which may provide a means to specifically target nanomedicine toward lesions. Moreover, a study with an induced pluripotent stem cell-derived in vitro MS BBB model implied the existence of intrinsic differences in BBB functioning between RRMS patients and healthy controls [379]. Thus, to improve the targeting of nanomedicine to MS lesions, future advances in MS treatment require the identification of targets at the MS BBB and knowledge of the (patient-specific) MS lesion microenvironment to identify stimuli that can be used to induce lesion-specific drug release. Alternatively, therapeutic agents, such as GD1a, that treat lesions while leaving healthy tissue unaffected may obviate the need for lesion-specific targeting.

Besides functional recovery, no optimal measure of remyelination in patients exists yet [380]. Therefore, in parallel, clinical biomarkers for remyelination should be developed and optimized to assess the effectiveness of (remyelination) therapy. MRI is currently not a reliable measure of remyelination [381]. Instead, advanced MRI techniques, such as diffusion tensor imaging, which measures the diffusion of water along axons, is particularly useful for the imaging of white matter tracts in the brain [381,382]. Other potential biomarkers or diagnostic tools include the plasma levels of neurofilament light chains [383], position emission tomography tracers for myelin [384], and multifocal visually evoked potential [385]. As of yet, none of these techniques have the sensitivity required to assess the remyelination of individual MS lesions. Nonetheless, they may still provide benefits for the global assessment of remyelination therapy effectivity. An interesting development is the use of magnetic resonance elastography (MRE), which measures the mechanical properties of tissue [386,387]. Brain viscoelasticity is reduced in MS [388,389] and appears to correlate with demyelination and ECM degradation in the cuprizone model [390] and inflammation in EAE [391]. Advantageous to MRE is that it allows for in vivo, localized imaging of inflammatory lesions. Interestingly, reduced brain viscoelasticity correlates with the upregulated expression of cellular Fn during inflammation in EAE [392]. Nevertheless, even though MRE correlates with de- and remyelination in experimental models [390], it is not a direct measure of remyelination, nor has it been established as a direct measure of de- and remyelination in MS. Moreover, it was recently observed that MRE measures of demyelinated white matter and normal-appearing white matter did not differ [393]. Thus, the search for a reliable biomarker or diagnostic tool for the detection of remyelination in patients is still ongoing and will require further optimization of existing techniques or the development of new techniques in the future.
